# Protein-Ligand Identification and *In Vitro* Inhibitory Effects of Cathine on 11 Major Human Drug Metabolizing Cytochrome P450s

**DOI:** 10.1177/10915818221103790

**Published:** 2022-06-04

**Authors:** Sharoen Y. M. Lim, Jason Siau Ee Loo, Mustafa Alshagga, Mohammed A. Alshawsh, Chin E. Ong, Yan Pan

**Affiliations:** 1 Division of Biomedical Sciences, School of Pharmacy, 69861 University of Nottingham Malaysia, Semenyih, Malaysia; 2 Centre for Drug Discovery and Molecular Pharmacology, Faculty of Health and Medical Sciences, 65214 Taylor’s University, Selangor, Malaysia; 3 Department of Pharmacology, Faculty of Medicine, 37447 Universiti Malaya, Kuala Lumpur, Malaysia; 4 School of Pharmacy, 50103 International Medical University, Kuala Lumpur, Malaysia

**Keywords:** cathine, CYP, *in vitro*, docking, herb-drug interaction

## Abstract

Cathine is the stable form of cathinone, the major active compound found in khat (*Catha edulis Forsk*) plant. Khat was found to inhibit major phase I drug metabolizing cytochrome P450 (CYP) enzyme activities *in vitro* and *in vivo*. With the upsurge of khat consumption and the potential use of cathine to combat obesity, efforts should be channelled into understanding potential cathine-drug interactions, which have been rather limited. The present study aimed to assess CYPs activity and inhibition by cathine in a high-throughput *in vitro* fluorescence-based enzyme assay and molecular docking analysis to identify how cathine interacts within various CYPs’ active sites. The half maximal inhibitory concentration (IC_50_) values of cathine determined for CYP2A6 and CYP3A4 were 80 and 90 μM, while CYP1A2, CYP2B6, CYP2C8, CYP2C9, CYP2C19, CYP2D6, CYP2E1, CYP2J2 and CYP3A5 showed no significant inhibition. Furthermore, in K_i_ analysis, the Lineweaver-Burk plots depicted non-competitive mixed inhibition of cathine on both CYP2A6 and CYP3A4 with K_i_ value of 63 and 100 μM, respectively. Cathine showed negligible time-dependent inhibition on CYPs. Further, molecular docking studies showed that cathine was bound to CYP2A6 via hydrophobic, hydrogen and π-stacking interactions and formed hydrophobic and hydrogen bonds with active site residues in CYP3A4. Both molecular docking prediction and *in vitro* outcome are in agreement, granting more detailed insights for predicting CYPs metabolism besides the possible cathine-drug interactions. Cathine-drug interactions may occur with concomitant consumption of khat or cathine-containing products with medications metabolized by CYP2A6 and CYP3A4.

## Introduction

Cathine (d-norpseudoephedrine (NPE)) is one of the major constituents found in the *Catha edulis Forsk* plant, which is also commonly known as khat.^1^ Khat is a perennial shrub cultivated in khat-belt countries such as in Africa and the Middle East, that are ingested not to gain nutritive values but to attain psychostimulatory effects.^2^ Khat was traditionally used in social gatherings in Yemen and widely cultivated as a source of income replacing coffee cultivation despite debates that khat cultivation drains foreign investment.^3^ Khat chewing is a tradition in khat-belt countries with each khat sessions lasting 3 to 4 hours with 100-200 g of leaves chewed, which induces mild euphoria and excitation in its users.^4^ In the early 1930s, Wolfes identified cathine (S,S(+)phenylpropanolamine) as norpseudoephedrine, which was one of the khat alkaloids that contribute to the pharmacological effects of khat leaves.^5^ It was reported that in every 100 g of fresh khat, there were 36 mg of cathinone, 120 mg of cathine and 8 mg of norephedrine present.^5^ Young stems and flowers of khat plants contain 1-phenyl-1,2-propanedione, cathinone, cathine and norephedrine.^6^ Cathinone reductase present in khat was accountable for reducing cathinone to cathine in the presence of NADPH.^6^ However, the quantity of cathine depends on the type of khat in which green khat contains a higher amount of cathine and norephedrine than red khat.^6^ Besides khat plant, cathine is also found in anorectic products.^7^ Cathinone and cathine shares closely similar chemical structures to amphetamine.^8^ Cathine was claimed to be 7-10 times less potent than cathinone but possesses a longer duration of action.^9^

Over the past decades, cathine has received little experimental attention. Cathine acts as a central stimulant (indirect sympathomimetic) and an inhibitor or monoamine oxidase.^7^ As compared to cathinone, cathine has a slower onset of action and may not be as lipophilic to enter the central nervous system, which to some extent explains the slower metabolism of cathine.^10^ Oral administration of cathine (6.5 mg/kg) may affect adrenocortical function and was found to produce rapid synchronized cell death in human leukaemia cell lines and peripheral blood leucocytes.^10^ With its milder psychostimulatory effects, a higher dose of cathine is needed to exert its effects, and this causes severe adverse systemic effects and the inhibition of noradrenaline uptake.^11,12^ In recent years, khat use was reported in England, Wales, Rome, Amsterdam, Canada, Israel, Australia, New Zealand and the United States.^13^ Khat is chewed by individuals idling on streets in Europe accompanied sometimes by alcoholic beverages and other drugs.^3^

It was found that a majority of khat users used more than one other psychoactive substances in which cigarettes were mainly used to maximize the stimulation power of khat with a combination of alcohol to break the aftereffect.^14^ Polysubstance users are also found to be using synthetic cathinone^15^ and cathine concurrently with alcohol leading to haemorrhage and intoxication.^16^ Moreover, khat use have been found to hinder antipsychotic^17^ and tuberculosis^18^ medication effects on patients who are khat users. With the widespread global use of khat together with the co-administration with clinically used drugs, the detrimental effects of khat-drug interactions are pushed towards the centre of the attention. According to the National Drug Intelligence Center, a component of the U.S. Department of Justice, the potency of harvested khat fades after 48hours as cathinone degrades into cathine (https://www.justice.gov/archive/ndic/pubs31/31482/index.htm). Thus, the effects of cathine on human drug metabolizing enzymes and the possible herb-drug interactions caused by cathine warrant further exploration.

Cytochrome P450 (CYP) are a crucial family of enzymes for producing cholesterol, steroids, prostacyclins and thromboxane A2 besides playing an essential role in detoxification of foreign substances and drugs.^19^ CYPs are predominantly expressed in the liver, besides also occurring in the small intestines, lungs, placenta and kidneys. In mammals, CYP family 1, 2, 3 and 4 are involved in detoxification, steroid and eicosanoids metabolism.^20^ In humans, CYP family 1, 2, 3 and 4 and to a lesser extent CYP family 5, 8, 19, 21 and 26 are involved in xenobiotic metabolism.^20^ The CYP enzymes are predisposed to inhibition or induction by xenobiotics including herbal medications that comprise mixtures of phytochemicals.^21^ Phytochemicals such as resveratrol and quercetin have been reported to significantly inhibit CYP3A4 which translates to reduced clearance and subsequent toxicity from other CYP3A4 substrates in humans.^22,23^ There are few well characterized examples including resveratrol and quercetin which were classified as clinically significant perpetrators as numerous herbal preparations interact with CYPs *in vitro* but showed variable *in vivo* interactions.^24^

Investigations into the inhibitory effects of cathine on CYPs are generally lacking in the literature as most studies about khat-drug interactions have focused on cathinone, which is deemed to be the major active compound in khat. Our earlier study found that khat ethanol extract (KEE) inhibited CYP2C9, CYP2D6 and CYP3A4 significantly, but cathinone showed negligible inhibition on these CYP isoforms *in vitro*,^25^ suggesting that other active constituents in the khat plant such as merucathine, merucathinone, tannins, cathedulins and norephedrine were likely responsible for these inhibitions. Ensuing this study, it was found that KEE also inhibited other major human drug metabolizing CYP isoforms namely CYP2A6, CYP2B6, CYP2C8, CYP2C19, CYP2E1, CYP2J2 and CYP3A5 except CYP1A2 *in vitro.*^26^ Our ongoing studies, hence, are investigating modulatory effects of main active compounds of khat on the major drug metabolizing CYPs *in vitro*. The current study aimed to follow up on the *in vitro* inhibitory effects and mode of inhibitions of cathine, which is the second main active component present in khat plant, on several major drug metabolizing CYPs namely CYP1A2, CYP2A6, CYP2B6, CYP2C8, CYP2C9, CYP2C19, CYP2D6, CYP2E1, CYP2J2, CYP3A4 and CYP3A5. Additionally, molecular docking was performed to understand how cathine interacts with particular CYP isoforms for which its *in vitro* results showed significant inhibition.

## Materials and Methods

### Chemicals and Reagents

Cathine or d-Cathine.HCl (d-Norpseudoephedrine.hydrochloride; (*1 S*, *2 S*)-2-amino-1-phenylpropan-1-ol.hydrochloride) was acquired from Lipomed AG (Arlesheim, Switzerland). Acetonitrile was obtained from Fisher Scientific (Loughborough, Leicestershire, UK). The Vivid^®^ CYP450 Screening kits for all CYP isoforms namely CYP1A2, CYP2A6, CYP2B6, CYP2C8, CYP2C9, CYP2C19, CYP2D6, CYP2E1, CYP2J2, CYP3A4 and CYP3A5 were obtained from Life Technologies™ (Carlbad, CA, USA). Costar Black 96-well plates and tris-base powder were obtained from Thermo Fisher Scientific (Pittsburg, PA, USA) and Amresco^®^ LLC (Solon, Ohio, USA), respectively.

### Determination of CYPs Activity Using Vivid P450 Assay Kits and Time Curve

Fluorescence readings (RFU) produced by blue (3-cyano-7-hydroxycoumarin), green (fluorescein) and cyan (7-hydroxy-4-trifluoromethylcoumarin) standards against a range of concentrations of the respective standard was plotted to derive the standard curves. The standard curve equation was used in succeeding assays to enumerate the fluorescent metabolites produced. Time curves were plotted afterwards to define the incubation time for single CYP assays namely 60 minutes for CYP1A2, CYP2A6, CYP2B6, CYP2C8, CYP2C9, CYP2C19, CYP2D6, CYP2E1, CYP2J2, CYP3A4 and 120 minutes for CYP3A5, respectively, as determined previously.^25,26^

The inhibitory effects of cathine on human CYP enzymes activities were determined using Vivid^®^ CYP450 Screening Kits^27^ for all CYP isoforms including Vivid^®^ EOMCC CYP1A2 Blue, Vivid^®^ CC CYP2A6 Blue, Vivid^®^ BOMCC CYP2B6 Blue, Vivid^®^ DBOMF CYP2C8 Green, Vivid^®^ BOMCC CYP2C9 BLUE Vivid^®^ EOMCC CYP2C19 Blue, Vivid^®^ EOMCC CYP2D6 BLUE Vivid^®^ EOMCC CYP2E1 Blue, Vivid^®^ MOBFC CYP2J2 Cyan Vivid^®^ BOMCC CYP3A4 BLUE and Vivid^®^ BOMCC CYP3A5 Blue, according to manufacturer’s instructions. Using the 96-well black plates, in each well, 40 μL of respective reaction buffers (Buffer I for CYP1A2, CYP2B6, CYP2D6, CYP3A4, CYP3A5; Buffer II for CYP2A6, CYP2C8, CYP2C9, CYP2C19, CYP2J2; Buffer III for CYP2E1) was added with 50 μL of master premix (including CYP450 BACULOSOMES^®^ Plus, human CYP reductase, potassium phosphate buffer, NADPH regeneration system containing glucose-6-phosphate buffer (333 mM) and 0.3 μ/mL glucose-6-phosphate dehydrogenase in 100 mM potassium phosphate at pH 8.0) for incubation with shaking at room temperature for 30 minutes. 10 μL per well of a mixture of respective substrates and NADP^+^ were added to kick start the reaction. Total volume per well was 100 μL in the 96-well plate.

The CYP enzyme specific substrates added were as follows: Vivid^®^ BOMCC (7-benzyloxymethyloxy-3-cyanocoumarin) for CYP2B6, CYP2C9, CYP3A4 and CYP3A5; Vivid^®^ CC (3-cyanocoumarin) for CYP2A6; Vivid^®^ DBOMF (dibenzylmethylfluorescein) for CYP2C8; Vivid^®^ EOMCC (ethoxymethyloxy-3-cyanocoumarin) for CYP1A2, CYP2C19, CYP2D6 and CYP2E1; Vivid^®^ MOBFC (7-p-methoxy-benzyloxy-4-trifluorocoumarin) for CYP2J2; and 0.03 mM NADP^+^. The mixture was shaken at room temperature, 60 minutes for CYP1A2, CYP2A6, CYP2B6, CYP2C8, CYP2C9, CYP2C19, CYP2D6, CYP2E1, CYP2J2, CYP3A4 and 120 minutes for CYP3A5. 50 μL of 0.5 M tris-base solution was added lastly to halt the reaction. The enzyme activities were measured by using the Varioskan^®^ Fluorescence Spectrophotometer (Thermo Fisher Scientific^®^, Waltham, MA, USA) at excitation/emission wavelengths of 415/460 nm (blue – CYP1A2, CYP2A6, CYP2B6, CYP2C9, CYP2C19, CYP2E1, CYP2D6, CYP3A4 and CYP3A5), 415/520 nm (cyan – CYP2J2) and 490/520 nm (green – CYP2C8).

### Reversible Inhibition

Cathine was dissolved in water to attain the 2.5 mM of cathine stock. The assay conditions were as described above. The assay conditions were as described above, with the exception that 40 μL of buffer was substituted using 40 μL of cathine with concentrations ranging from 0 to 1000 μM, which was obtained following two times serial dilution of the cathine stock. Cathine inhibited CYP2A6 and CYP3A4 according to their IC_50_ values which were less than 100 μM, and then their K_i_ values were further assessed. Various concentrations of cathine (0, 31.25, 62.5, 125, 250, 500, 1000 μM) were incubated with Vivid^®^ Fluorogenic Probe Substrates CC (5, 10, 20, 40 μM) for CYP2A6 and BOMCC (5, 10, 20, 40 μM) for CYP3A4.

### Determination of Time-Dependent Inhibition

In mechanism-based or time-dependent inhibition, the master premix was added with NADP^+^ to produce NADPH during the pre-incubation. The mixture (containing CYP450 BACULOSOMES^®^ Plus, human CYP reductase, potassium phosphate buffer, NADPH regeneration system and NADP^+^) was incubated for 30 minutes with shaking under similar range of concentrations of cathine as mentioned above for IC_50_ determination. Substrates were subsequently added to initiate the reaction, and fluorescence readings were taken after 60 minutes for CYP1A2, CYP2A6, CYP2B6, CYP2C8, CYP2C9, CYP2C19, CYP2D6, CYP2E1 and CYP2J2, CYP3A4 and 120 minutes for CYP3A5 incubation. The IC_50_ shift was derived by calculating ratios of IC_50_ obtained from pre-incubation with and without NADPH. IC_50_ shift ratio >2 implies irreversible time-dependent inhibition.^28^

### Data Analysis

To determine IC_50_ values, the remaining enzyme activity of each cathine concentration was divided by the solvent control well (without cathine but replaced by water) and multiplied the value with 100% to obtain the percent control activity (%) for each cathine concentration. The resulting percent control activity (%) were plotted against different concentrations of cathine to attain the half maximal inhibitory concentrations (IC_50_) curve. IC_50_ values of reversible and irreversible inhibition were determined by non-linear regression analysis using GraphPad Prism 9 for Windows (GraphPad Software, San Diego, CA). Data for inhibition constant (K_i_) analysis and mode of inhibition were evaluated using Excel spreadsheet (Microsoft, USA) and Lineweaver-Burk plots. The secondary plots of cathine concentrations against slopes of Lineweaver-Burk plots were plotted to determine the K_i_ values. All assays were carried out in triplicate and stated as mean ± SD.

### Molecular Docking

The crystal structures of human cytochrome P450 CYP2A6 (PDB code: 2FDV) and CYP3A4 (PDB code: 4D75) were retrieved from Protein Data Bank.^29^ Cathine conformations were generated using Open Babel GUI v2.3.1.^30^ The protein was prepared using AutoDock Tools 1.5.6 while AutoDock 4.2 software^31^ was used to carry out molecular docking. AutoGrid was used to create the grid box around co-crystallized ligand covering the active site of CYPs, which included the haem group. The grid size for specifying the search space was set at 60 × 60 × 60 Å (for CYP3A4) and 70 × 70 × 70 Å (for CYP2A6) with a default grid point spacing of 0.375 Å with a total of 10 docking runs. Docking simulations were performed using the Lamarckian genetic algorithm with 25 × 10^5^ energy evaluations and 27 000 iterations per run. The best configuration and possible ligand binding was then determined based on the binding score. After docking, analysis of the ligand interactions at the binding site was performed and visualized using the PyMOL Molecular Graphics system (Schrödinger, LLC, New York, NY, USA).

## Results

### Standard Curves and Time Curve

Standard curves were plotted using fluorescence readings (RFU) against standard concentrations, and the following standard equations and *R*^2^ values are obtained: (i) blue (3-cyano-7-hydroxycoumarin) standard with Buffer I, II and III, y = 0.0083x + 0.012, *R*^2^ = 0.9995, y = 0.0063x + 0.0132, *R*^2^ = 0.9983, y = 0.0093x + 0.0149, *R*^2^ = 0.998, respectively, (ii) cyan (7-hydroxy-4-trifluoromethylcoumarin) standard with Buffer II, y = 0.0003x + 0.0005, *R*^2^ = 0.9811 and (iii) green (fluorescein) standard to obtain the standard equations and *R*^2^ values, y = 0.0051x + 0.0029, *R*^2^ = 0.9978. Linear ranges of CYP1A2, CYP2A6, CYP2B6, CYP2C8, CYP2C19, CYP2E1 and CYP2J2 were from 0 – 60 minutes except 0 – 120 minutes for CYP3A5, and therefore, the incubation time was 1 hour for all CYP isoforms mentioned except 2 hours for CYP3A5 as determined in previous studies.^26^

### Inhibition of Cathine on CYPs

**Figure 1.**
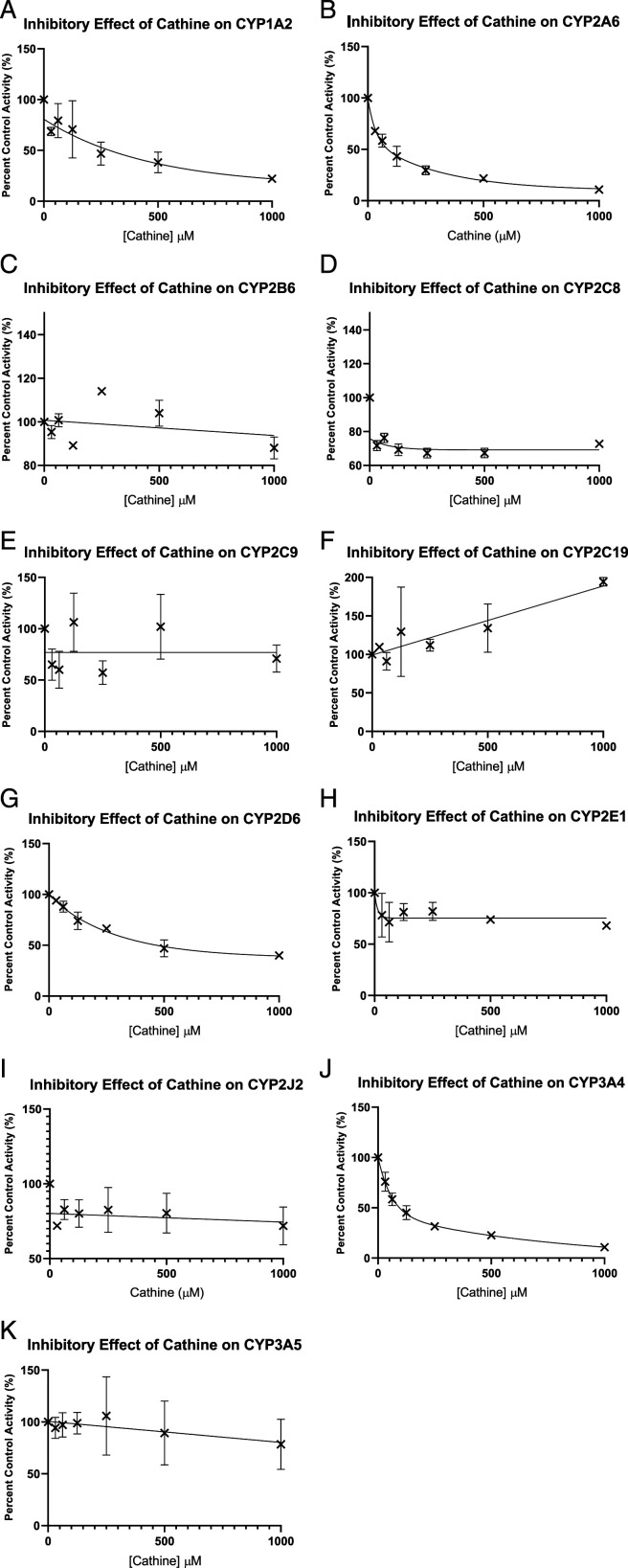
Inhibitory effects of cathine on (A) CYP1A2, (B) CYP2A6, (C) CYP2B6, (D) CYP2C8, (E) CYP2C9, (F) CYP2C19, (G) CYP2D6, (H) CYP2E1, (I) CYP2J2, (J) CYP3A4 and (K) CYP3A5. IC_50_ values were determined by non-linear regression analysis using GraphPad Prism version 9 for Windows (GraphPad Software, La Jolla California, USA). Each point represents mean ± SD (n = 3). CYP: Cytochrome P450, IC_50_: 50% inhibitory concentration.

Cathine significantly inhibited CYP2A6 and CYP3A4 with IC_50_ values of 80 and 90 μM as shown in non-linear graphs (Figure 1). Cathine showed no inhibition on CYP1A2, CYP2B6, CYP2C8, CYP2C9, CYP2C19, CYP2D6, CYP2E1, CYP2J2 and CYP3A5 with IC_50_ values more than 100 μM.

Table 1 shows the determined IC_50_ without/with NADPH in pre-incubation and IC_50_ shift for CYP1A2, CYP2A6, CYP2B6, CYP2C8, CYP2C9, CYP2C19, CYP2D6, CYP2E1, CYP2J2, CYP3A4 and CYP3A5. All the IC_50_ shifts showed no significant TDI potency as all the IC_50_ shifts are not >2.^28^

**Table 1.** IC_50_ without/with NADPH in pre-incubation, and IC_50_ shift of CYP1A2, CYP2A6, CYP2B6, CYP2C8, CYP2C9, CYP2C19, CYP2D6, CYP2E1, CYP2J2, CYP3A4 and CYP3A5 with cathine.

CYP isoform	IC_50_ without NADPH (μM)	IC_50_ with NADPH (μM)	IC_50_ shift
CYP1A2	285	255	1.12
CYP2A6	80	70	1.14
CYP2B6	>100	>100	-
CYP2C8	>100	>100	-
CYP2C9	Not determined	Not determined	-
CYP2C19	Not determined	Not determined	-
CYP2D6	435	860	0.51
CYP2E1	Not determined	Not determined	-
CYP2J2	Not determined	Not determined	-
CYP3A4	90	Not determined	-
CYP3A5	>1000	Not determined	-

*not determined – the curve plateaus above the 50% half maximal inhibitory line.

### K_i_ Analysis and Mode of Inhibition

**Figure 2.**
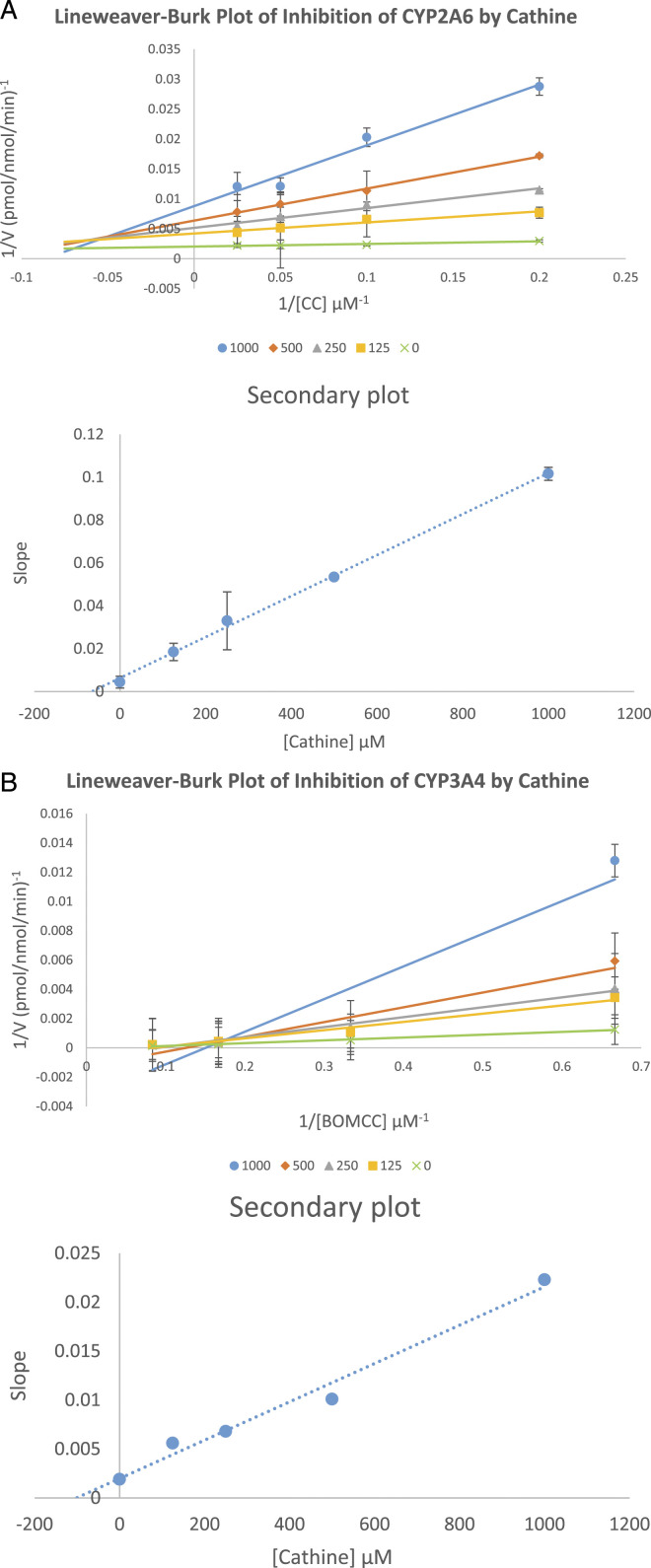
The Lineweaver-Burk plot or double reciprocal was plotted with inverse velocity (1/V) against the inverse of the substrate concentrations (1/[S]). Lineweaver-Burk plot of (A) inhibition of CYP2A6 by cathine and (B) inhibition of CYP3A4 by cathine at the indicated concentrations of cathine and substrate. The substrate concentration used were CC (5, 10, 20, 40 μM) for CYP2A6 and BOMCC (5, 10, 20, 40 μM) for CYP3A4. The secondary plots were plotted using slopes from Lineweaver-Burk plot against cathine concentrations which was used to derive the K_i_, inhibition constant values. Each data point are triplicates that was represented by mean ± SD (n = 3).

The K_i_ values were obtained from secondary plots of each CYP isoforms. Cathine inhibited CYP2A6 and CYP3A4 via non-competitive or mixed mode with K_i_ of 63 μM, and non-competitive or mixed mode with K_i_ of 100 μM as shown in the Lineweaver-Burk plots (Figure 2).

### Docking of Cathine Into Human CYPs

**Figure 3.**
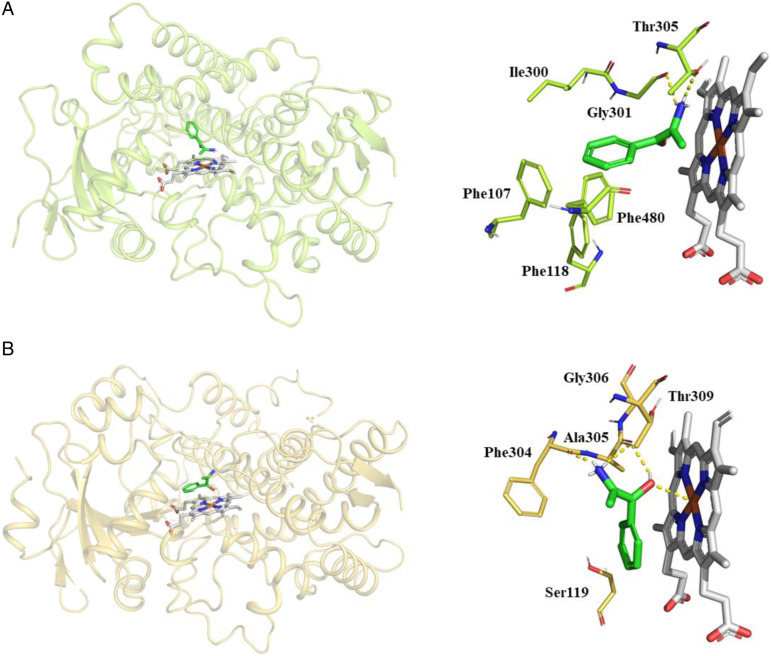
Molecular docking demonstrating binding modes and key interactions of cathine (green) relative to the haem group (white) in the active sites of (A) CYP2A6 (PDB 2FDV) and (B) CYP3A4 (PDB 4D75). Hydrogen bonds are displayed as yellow dashed lines. Non-polar hydrogens have been removed for visual clarity.

Cathine was docked into the active site of human CYP2A6 and CYP3A4. After inspection of the top-ranked poses in AutoDock, the potential binding sites were identified. The binding poses and key residues interacting with cathine are presented in Figure 3. The binding energies and the interacting residues for cathine with CYP2A6 and CYP3A4 are as shown in Table 2.

**Table 2.** Binding energies and interacting residues of cathine with CYP2A6 and CYP3A4.

CYP	Binding energy (kcal mol^−1^)	Amino acid residues	Interaction
CYP2A6	–7.96	Ile300, Thr305, Phe480	Hydrophobic
		Gly301, Thr305	Hydrogen bond
		Phe107, Phe118	π-stacking
CYP3A4	–6.53	Ser119, Ala305, Gly306, Thr309	Hydrophobic
		Phe304, Ala305	Hydrogen bond

## Discussion

Current knowledge on the mechanisms of action of khat and its active constituents with their short-term and long-term effects are not as extensive as other amphetamine-type stimulants.^32^ Khat use remains a debatable topic whether it is an innocuous cultural tradition or drug of abuse.^32^ Following the extensive use of khat with alcohol and clinically used drugs,^33^ there is an urgent obligation to explore the possible inhibitory effects of the second major active compound in khat, cathine, on human drug metabolizing CYP enzymes. *In vitro*, *in silico* and *in vivo* studies on cathine’s inhibitory effects on CYPs are generally lacking. Moreover, cathine emerged as an effective weight-lowering agent for adjunct treatment of obesity,^34^ but studies on cathine’s safety profile and efficacy is unmapped. The *in vitro* and *in silico* outcome from this study may contribute to the establishment of cathine’s safety profile.

With regard to herb-drug interaction via CYP inhibition by khat constituents, our previous study found that khat significantly inhibited CYP2D6, CYP2C9 and CYP3A4 while cathinone showed negligible inhibitory effects.^25^ Besides, the subsequent studies in our laboratory found that khat ethanol extracts inhibited most of the major drug metabolizing CYPs including CYP2A6, CYP2B6, CYP2C8, CYP2C19, CYP2E1, CYP2J2 and CYP3A5 except CYP1A2.^26^ On the other hand, our ongoing investigations demonstrated that the CYPs were inhibited by cathinone differently from that of khat extracts (unpublished data). A study by Bedada et al^35^ using human volunteers on one week of daily khat use (400 g) showed khat significantly inhibited CYP2D6, marginally inhibited CYP3A4, CYP2C19 and CYP1A2.^35^ An *in vivo* study using rats found that rats treated with both khat plus clopidogrel had significantly lower clopidogrel metabolite produced due to inhibition of CYP enzyme by khat.^36^ These studies supported that khat inhibited CYP activities but did not make any investigations on the specific compounds in khat that contributed to the CYPs inhibition. Therefore, it is plain to see that ingredients other than cathinone such as cathine and norephedrine, for instance, could be involved in the CYP inhibitory effects of khat.

Cathine showed no time-dependent (mechanism-based) inhibition on CYP1A2, CYP2A6, CYP2B6, CYP2C8, CYP2C9, CYP2C19, CYP2D6, CYP2E1, CYP2J2, CYP3A4 and CYP3A5 (Table 1). Cathine inhibited two CYPs out of the 11 CYP isoforms in this study (Table 2). Cathine inhibited CYP2A6 and CYP3A4 with IC_50_ values (Figure 2) of 80 and 90 μM, respectively. From the current *in vitro* findings, cathine inhibited both CYP2A6 and CYP3A4 via non-competitive or mixed mode with K_i_ values 63 μM and 100 μM, respectively, as shown in the Lineweaver-Burk plot (Figure 2). Non-competitive inhibition is this context would be cathine, as the inhibitor, may bind to an allosteric site which is a binding pocket other than the active site and thereby changes the overall shape of the site for normal substrate to prevent it from fitting, thus preventing reaction from taking place.^37^ Furthermore, mixed mode inhibition may also propose that cathine could be metabolized as a substrate or non-substrate.^38^ It is worth mentioning that an inhibitor could act as a substrate or non-substrate, and non-substrates have higher tendency to bind to allosteric site.^38^ These findings implied that cathine might affect the pharmacokinetics of drugs metabolized by CYP2A6 and CYP3A4 with respect to potential herb-drug interaction by inhibiting these two CYPs.

Based on our *in vitro* study, cathine, the compound which cathinone decomposes into after khat is harvested, appears to only significantly inhibit CYP2A6 and CYP3A4 out of the 11 CYP isoforms tested. CYP2A6 and CYP3A4 are abundantly expressed in the liver, with an expression rate of 3.8% and 12.0%, respectively.^39^ These CYPs played a major role in metabolizing various drugs. CYP2A6 metabolizes nicotine (the primary psychoactive component in cigarettes), cotinine, tegafur, letrozole, coumarin, methoxyflurane, efavirenz, valproic acid, pilocarpine, artemisinin, artesunate, caffeine, disulfiram, halothane, fadrozole and tyrosol,^40,41^ whereas CYP3A4 is responsible for 40% to 45% of all phase I metabolism and approximately 70% of gastrointestinal CYP activity beside being co-expressed with P-glycoprotein (PGP) in the liver and intestines.^42,43^ CYP3A4 metabolizes drugs such as paclitaxel, fentanyl, tamoxifen, tacrolimus and statins.^40^ Therefore, khat/cathine users who are on medications metabolized by CYP2A6 and CYP3A4 may experience herb-drug interactions or may fail to respond to some medications. Further human studies are warranted to investigate whether the inhibitory effects of khat/cathine on CYPs are clinically significant or otherwise.

To our best knowledge, pharmacokinetic studies on cathine are very limited. Khat chewing releases cathine and cathinone which was subsequently absorbed by the mucous membranes of the oral cavity and followed by the stomach lining.^44^ The average content of cathine in 100 g of fresh khat leaves is 83-120 mg^10^. A 100-400 g of fresh khat leaves is consumed on a daily basis.^45^ Hence, assuming that khat users who took 400 g of fresh khat leaves would have consumed about 332-480 mg of cathine, therefore, an average 70 kg adult with 5 L blood would have absorbed approximately 66.4 × 10^−3^ to 96 ×10^−3^ mg/ml of cathine or in other words, about 351.7-511.5 μM. Despite the low K_i_ values determined in our study that ranges from 63 to 100 μM, these values may be clinically relevant since khat users commonly take huge amount of khat on a daily basis. Toennes et al^45^ reported that at a mean of 43.8 g of khat chewed, the mean ingested dose of cathine in milligrams was 32.4, while the absorbed proportion of cathine ranges from 16% to 84%.^45^ The mucosa of the oral cavity is the first absorption segment, where the major fraction of the cathine (84 ± 6%) is absorbed.^45^ Cathine could be detected in the urine up to 50-70 hours after ingestion which was about a day time more than cathinone at 22-26 hours.^46^ In addition, a study found that cathine concentration in oral fluid was higher than that of cathinone because khat users did not chew fresh leaves and cathinone easily metabolized into cathine.^47^ According to European Monitoring Centre for Drugs and Drug Addiction, hepatic first-pass metabolism of cathinone forms norephedrine and only 2% of cathinone is excreted in the urine (https://www.emcdda.europa.eu/publications/drug-profiles/khat). The elimination half-life of cathine (5.2 ± 3.4 hours) is longer than cathinone (1.5 ± 0.8 hours) (https://www.emcdda.europa.eu/publications/drug-profiles/khat). As compared to cathinone, cathine is less lipophilic and may also penetrate to the central nervous system.^48^ Nevertheless, lipophilicity of cathine should also be considered during prescribing by healthcare professionals to khat users because lipophilicity impacts cellular uptake and ADMET (absorption, distribution, metabolism, excretion and toxicity).^49^ High dosage accompanied by high lipophilicity compound is a detrimental combination.^50^ Although less lipophilic than cathinone, the lipophilicity of cathine may still affect metabolic activity as lipophilic compounds showed greater affinity to metabolic enzymes. Khat compounds’ lipophilicity need to be known during drug administration as they may readily cross the blood brain barrier.^51^

However, in the early 1945-1961, there were contradicting debates by researchers that cathine has low potency and the amount of cathine in khat is insufficient to exert symptoms shown after consumption versus cathine was the only khat alkaloid responsible to give pharmacological significance.^4^ Dried khat leaves showed an increase in cathine amount from 0.172% to 0.192% (fresh khat) to 0.184-0.198%.^52^ According to one case report, a male patient who suffered from haemorrhagic stroke with hypertension was found to have no chronic diseases history but instead daily khat chewing.^53^ The patient’s urine samples showed positive for amphetamine-like substance while liquid chromatography-tandem mass spectrometry (LC-MS/MS) detected presence of cathine and cathinone. The serum concentration of cathine was 100-fold of cathinone, suggesting that the patient’s symptoms are most likely due to cathine.^53^ The maximal plasma concentrations of cathine will be reached at 2.6 hours with a mean residence time (MRT) of 10.2 ± 2.6 hours, which was twice more than cathinone.^45^ Cathine retain in the human body for a longer period of time as compared to cathinone and could be detected in the urine samples even after approximately ≥3 days after ingestion.^46^ Since cathine is retained for a longer period of time within the human body, it may exert greater inhibitory effects on human CYPs and higher chances of causing khat-drug interactions as compared to cathinone. Nevertheless, the effects of cathine retaining within the body for a longer time have yet to be explored.

From the molecular docking analysis, the interactions between cathine and the CYPs consisted of hydrogen bonds, hydrophobic and to a lower extent, π-π stacking (Table 2). As our *in vitro* findings showed non-competitive mixed mode of inhibition, our molecular docking analysis provided a prediction of the interaction of cathine with the active site of CYP2A6 and CYP3A4 which may contribute to the mixed mode inhibition component. Intermolecular interactions namely hydrogen bonding and hydrophobic interactions were also previously established to be the chief determinants in determining the stability of energetically favoured ligands within the binding pocket.^54^ Cathine interacted with CYP2A6 via four hydrophobic interactions with Ile300, Thr305, Phe480, hydrogen bonding with Gly301 and Thr305 and single π-stacking with Phe107 and Phe118 (Figure 3). Coumarin, a prototype substrate of CYP2A6, was reported to be more efficient in the hydroxylation process as Gly301 in CYP2A6 enables coumarin to adopt a pose that brings 7-hydroxylation site closer to the haem group.^55^ The higher number and stronger hydrogen bonds in CYP2A6-cathine complex offered a greater binding affinity of cathine to CYP2A6 as compared to CYP3A4-cathine complex. As in the case of CYP3A4 with cathine, there were four hydrophobic interactions with Ser119, Ala305, Gly306 and Thr309 and hydrogen bonding with Phe304 and Ala305 formed (Figure 3). Ala305 and Thr309 was also observed in interactions between ketoconazole, a potent inhibitor of CYP3A4/5, and inhibitors derived from seaweed namely morin, quercetin, fucoxanthin, siphonaxanthin and hesperidin^56^; this supports the notion that the binding of cathine to the active site of CYP2A6 and CYP3A4 is similar to other known inhibitors. The *in silico* comparison of CYP2A6 and CYP3A4 was also in agreement to the *in vitro* inhibition determined as cathine inhibited CYP2A6 (K_i_ = 63 μM) more strongly than CYP3A4 (K_i_ = 100 μM). This conclusion is warranted by the binding energy value of the CYP2A6-cathine complex of −7.96 kcal mol^−1^ that exceeds the binding energy value of CYP3A4-cathine complex of −6.53 kcal mol^−1^. Non-polar or hydrophobic interactions are known to play a major role in contributing to binding forces and folding stability of the proteins or in other words the CYP enzyme while charged residues interacting with polar groups in the form of hydrogen bonds reinforce specificity.^57^ Both cathine-CYP complexes showed similar hydrogen bonds on one end of the cathine molecule (Figure 3). However, a cluster of phenylalanine residues are present in CYP2A6 that interacts with and stabilizes the aromatic ring of cathine. Conversely, there are less nearby residues interacting between cathine and CYP3A4, indicating that CYP3A4 could have a more open active site. These factors may contribute to the lower predicting binding free energy with CYP2A6 and the correspondingly lower K_i_ value.

We have performed docking using known inhibitors of CYP2A6 (PDB 2FDY) – aldrithiol and CYP3A4 (PDB 2V0M) – ketoconazole from Protein Data Bank to compare with cathine’s interactions. Cathine overlaps well with experimentally determined binding modes of aldrithiol and ketoconazole (at the same position above the haem group of CYP2A6 and CYP3A4 – as shown in Supplemental Figure 1). In CYP2A6, cathine overlaps very well with aldrithiol while ketoconazole is much bigger in size and has more interactions as compared to cathine in CYP3A4. In the active site of CYP2A6, aldrithiol formed hydrogen bonds with Asn297, π-stacking with Phe107, Phe111, and Phe118 and hydrophobic bonds with Phe209, Ile300, Gly301, Thr305 and Phe408. Ketoconazole formed π-stacking with Phe304, and strong ionic interaction with Fe^2+^ of the haem group of CYP3A4 and hydrophobic bonds with Phe57, Arg105, Ser119, Leu210, Phe241, Ile301, Ala305, Ala370, Arg372, Glu374, Gly481 and Leu482. Some similar interactions seen from our docking outcome such as (1) in CYP2A6, cathine and aldrithiol (4,4′-Dipyridyl disulfide) both have a series of π-stacking interactions with Phe107, Phe118 and Phe480 to stabilize them, hydrophobic interactions with Thr305^58^ and (2) in CYP3A4, cathine and ketoconazole both have π-stacking with Phe304 formed.^59^

The current study utilizes high-throughput fluorescence-based assays for detection of enzyme-drug interactions.^26,60^ In spite of that, a number of caveats exist in this *in vitro* study using recombinant enzyme preparations as compared to human liver microsomes or hepatocytes. In real-life, owing to the different non-specific binding, accessory proteins or protein-protein interactions, the enzyme matrix itself may contribute to prominent variations in IC_50_ values from one *in vitro* system to the other^61^ which could not be investigated using the current *in vitro* methods. Moreover, fluorescent substrates may interact with CYPs differently than classic drug substrates in the body.^62^ Besides that, gastric digestion, absorption of cathine and oral administration first-pass effect also could not be included using the current *in vitro* screening tool.^62^ In addition, the present model system cannot predict enzyme induction, which occurs upon ingestion to some botanicals and their derivatives. The current *in vitro* method is a simple model, and therefore, further studies should encompass relevant drug substrates with liver microsomes or hepatocytes.^62^ The high flexibility of the CYP active site poses another challenge to our docking analysis to accurately predicting the appropriate substrate binding modes and also the site of metabolism (SOM) of the substrate (e.g. cathine).^63^ Further studies should employ docking combined with molecular dynamics simulations to investigate effects of SOM flexibility of CYPs. The *in vitro* mixed mode inhibition results have raised another limitation of this study especially when mixed mode of inhibition suggests multiple possible binding modes but could not be predicted from our docking study. What is more, the allosteric sites of CYP enzymes are an area of active research, although locations among helices C, E and H have recently been proposed.^64^

It is worth mentioning that *in vitro* and docking predictions are lacking in biotransformation capabilities,^65^ and the lack of *in vivo* biological mechanisms has probably stronger impact on the accuracy of toxicity evaluations as cathine inhibitory effects on CYPs were performed *in vitro*.^66^ This study answers some previous questions regarding the inhibitory effects of cathine on major human drug metabolizing CYP enzymes but also raises new questions to explore. As mentioned earlier that cathine is retained in the human body for a longer period of time as compared to cathinone, therefore future studies should consider effects of cathine on CYP1A1 that are present in large amount in gut cells, shedding light to enteric drug interaction potential. Research has also shown that (1R)(2S)-norephedrine was not the final product produced from the phenypropylamino alkaloids pathway in khat plant as claimed in earlier studies, but instead the metabolic pathway continues downstream of cathine to form oxazolidine derivates.^67^ Therefore, future studies are recommended to look into the inhibitory effects *in vitro* and *in silico* of other khat constituents including norephedrine and oxazolidine derivatives. This area of research remains widely unexplored, giving room for further research on the unique phytochemistry of khat to better understand the khat-drug interactions besides detecting possible similar phytoconstituents that are also present in other herbal products, thus indirectly helping in understanding therapeutic potential of other herbs as well. Future *in vivo* studies using human subjects and animal models are also warranted to look into the effect of specific khat contents, for instance, cathinone, cathine, norephedrine, merucathine, merucathinone, cathedulins and tannins.

## Conclusion

Cathine reversibly inhibited CYP2A6 and CYP3A4 activities. Docking results of CYP2A6 and CYP3A4 is aligned to the *in vitro* inhibition determined as cathine inhibited CYP2A6 with K_i_ value of 63 μM which was stronger than CYP3A4 with K_i_ value of 100 μM. This deduction is justified by the binding energy value of the CYP2A6-cathine complex of −7.96 kcal mol^−1^ that exceeds the binding energy value of CYP3A4-cathine complex of −6.53 kcal mol^−1^. Additional π-stacking with nearby phenylalanine residues in CYP2A6 may explain why cathine showed more stable and stronger affinity on CYP2A6 than CYP3A4. In silico, *in vitro* and in vivo research on cathine are scarcely explored, and this study offers useful insights into the molecular interaction of cathine with CYPs besides directing future studies in the khat-drug interaction area. This study is a preliminary screening that must be validated by means of various approaches. Nevertheless, in view of the budding khat and cathinone derivatives’ use, knowledge on khat-drug, cathinone-drug or cathine-drug interactions are urgently needed. The current *in vitro* preliminary findings of this study require more diverse models (e.g. liver microsomes, hepatocytes, *in vivo* animal or human studies) before a comprehensive exploration in clinical trials. The current study showed synergistic results from both *in vitro* and *in silico* docking evaluation of cathine as the inhibitor of CYPs enzyme activities. Our study proved that cathine exhibited negligible *in vitro* inhibitory effects on CYP1A2, CYP2B6, CYP2C8, CYP2C9, CYP2C19, CYP2D6, CYP2E1, CYP2J2 and CYP3A5, and significant inhibition on CYP2A6 and CYP3A4. Despite this preliminary *in vitro* screening outcome, it would be best to recommend healthcare experts and patients to not co-administer khat or cathine-containing products with clinical drugs to minimize the possible cathine-drug interactions, especially those metabolized by CYP2A6 and CYP3A4.

## Supplemental Material

Supplemental Material - Protein-Ligand Identification and *In Vitro* Inhibitory Effects of Cathine on 11 Major Human Drug Metabolizing Cytochrome P450s Click here for additional data file. Supplemental Material for Protein-Ligand Identification and *In Vitro* Inhibitory Effects of Cathine on 11 Major Human Drug Metabolising Cytochrome P450s by Sharoen Y. M. Lim, Jason Siau Ee Loo, Mustafa Alshagga, Mohammed A. Alshawsh, Chin E. Ong, and Yan Pan in International Journal of Toxicology
