# Supplementary material for: Protein-Ligand Identification and In Vitro Inhibitory Effects of Cathine on 11 Major Human Drug Metabolizing Cytochrome P450s
Source: Int J Toxicol. 2022 Jun 4;41(5):355–66. doi: 10.1177/10915818221103790 (PMC9411691; doi:10.1177/10915818221103790)
Supplement: Supplemental Material - Protein-Ligand Identification and In Vitro Inhibitory Effects of Cathine on 11 Major Human Drug Metabolizing Cytochrome P450s [file sj-pdf-1-ijt-10.1177_10915818221103790.pdf]

Supplementary materials

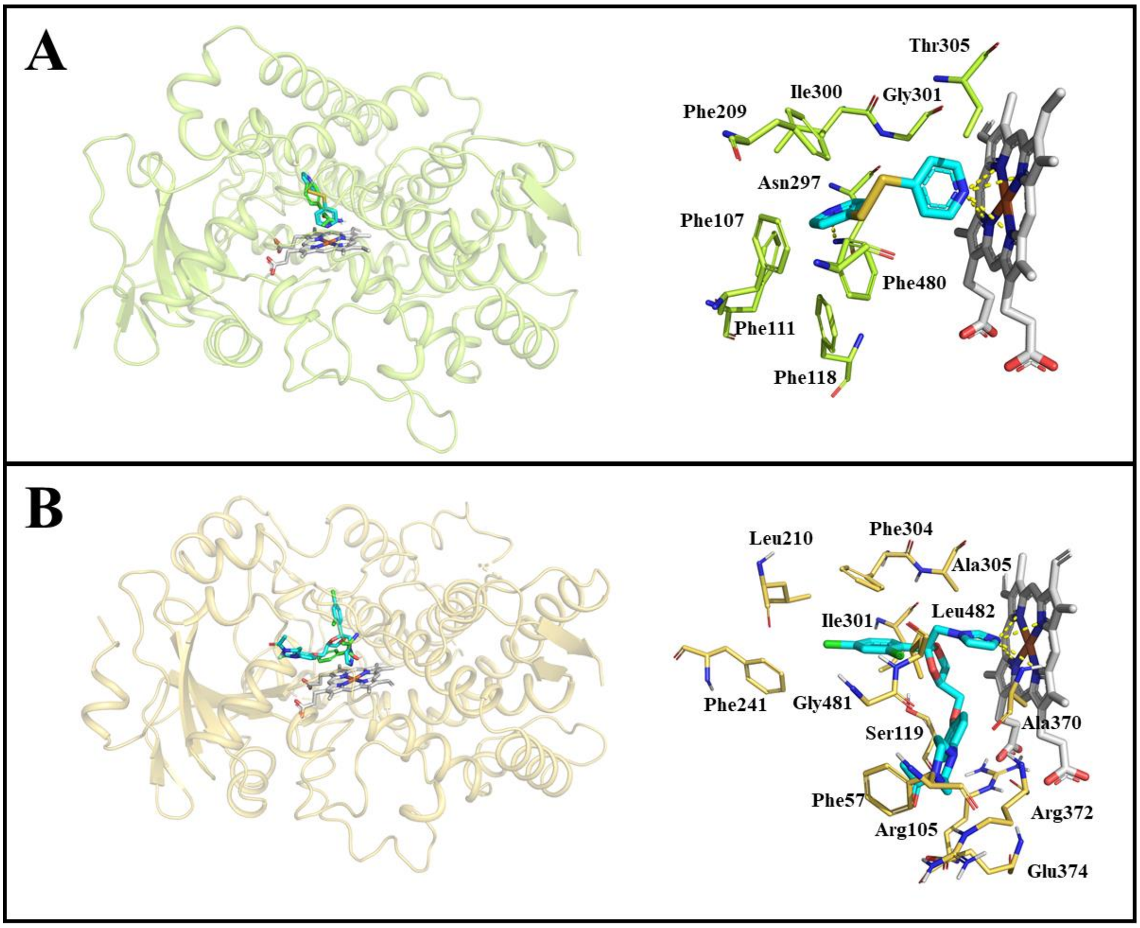

Supplementary Fig 1. Molecular docking (on the left) demonstrating binding modes and key interactions of cathine (green) overlapped with adrithiol or ketoconazole (cyan) relative to the haem group (white) in the active sites of (A) CYP2A6 (PDB 2FDY) and (B) CYP3A4 (PDB 2V0M). The right side shows the key interactions for adrithiol and ketoconazole. Hydrogen bonds are displayed as yellow dashed lines. Non-polar hydrogens have been removed for visual clarity.
